# Thiazine Red^+^ platelet inclusions in Cerebral Blood Vessels are first signs in an Alzheimer’s Disease mouse model

**DOI:** 10.1038/srep28447

**Published:** 2016-06-27

**Authors:** Kathrin M. Kniewallner, Daniela Wenzel, Christian Humpel

**Affiliations:** 1 Laboratory of Psychiatry and Exp. Alzheimer’s Research, Department of Psychiatry Psychotherapy and Psychosomatik, Medical University of Innsbruck, Austria; 2 Institute of Physiology I, University Hospital Bonn, Germany

## Abstract

Strong evidence shows an association between cerebral vascular diseases and Alzheimer´s disease (AD). In order to study the interaction of beta-amyloid (Aβ) plaques with brain vessels, we crossbred an AD mouse model (overexpressing amyloid precursor protein with the Swedish-Dutch-Iowa mutations, APP_SweDI) with mice expressing green fluorescent protein (GFP) under the flt-1/VEGFR1 promoter in vessels (GFP_FLT1). Our data show, that only very few Aβ plaques were seen in 4-months old mice, focused in the mammillary body and in the lateral septal nucleus. The number of plaques markedly increased with age being most prominent in 12-months old mice. Thiazine Red was used to verify the plaques. Several Thiazine Red^+^ inclusions were found in GFP^+^ vessels, but only in non-perfused 4-months old mice. These inclusions were verified by Resorufin stainings possibly representing cerebral amyloid angiopathy. The inclusions were also seen in non-crossbred APP_SweDI but not in wildtype and GFP_FLT1 mice. In order to characterize these inclusions Flow Cytometry (FACS) analysis demonstrated that platelets were specifically stained by Thiazine Red^+^, more pronounced when aggregated. In conclusion, our data show that Thiazine Red^+^ inclusions representing aggregated platelets are a first pathological sign in AD before plaque development and may become important therapeutic targets in early AD.

Alzheimer’s disease (AD) is the most common form of dementia in the elderly and leads to progressive impairment of memory and cognitive decline^1^. The disorder is characterized by accumulation of beta-amyloid (Aβ) in brain (plaques) and blood vessels (cerebral amyloid angiopathy, CAA), Tau pathology, inflammatory processes and cerebrovascular dysfunction. The causes for developing AD are not well known, but the Aβ cascade^2^ is the major hypothesis. However, whether the presence of Aβ plaques directly causes AD or if Aβ plaques are generated secondary to AD is still an ongoing question. Furthermore the presence of cerebrovascular diseases is considered to be a criterion for AD^3^. Cerebrovascular dysfunction occurs in AD patients, leading to alterations in blood flow that might play an essential role in the pathogenesis of AD^4^.

There is more and more evidence that cerebrovascular dysfunction might be an early event in the pathogenesis of AD. Interestingly, cerebrovascular dysfunction with alterations in cerebral blood flow has emerged as a potent predictor of AD^3^. Hence, vascular dysfunction may play a critical role in AD, because cerebral hypoperfusion and impaired Aβ clearance across the blood-brain barrier (BBB) may contribute to the onset of AD. Subsequently, impaired clearance of Aβ from the brain may lead to accumulation of Aβ in blood vessels and in brain parenchyma resulting in CAA. Finally, these vessel depositions may disrupt the integrity of the blood vessel wall^5^. Interestingly, there are indications that every Aβ plaque is either in direct contact with or closely related to a small vessel^6^.

CAA is a key pathological sign in AD and is caused by deposition of Aβ in the walls of vessels in the cerebral cortex and leptomeninges, causing vessel rupture, cerebral hemorrhage and microbleeds^7^. Therefore, CAA is seen as an age–associated disease of the elderly and in patients with AD^8^. The generation of CAA is not clear, but vascular risk factors, such as e.g. hypoperfusion or reduced vascular autoregulation may cause vascular bleeding and subsequent influx of toxic molecules into the brain. It is hypothesized that blood cells, especially platelets may contribute to the formation of CAA, because platelets (1) play a role in repair of damaged blood vessels and (2) platelets contain high amounts of amyloid precursor protein (APP) and produce predominantly Aβ_40_^9^,^10^. It seems reasonable that an initial deposition of Aβ in early stages of CAA may induce degeneration of the vessel wall leading to dilation of the lumen^11^. However, to date it is not clear if CAA is a primary cause in the development of AD or only a consequence of the Aβ accumulation in the brain^12^.

In order to study the development of Aβ plaques and the association with brain vessels especially the formation of CAA, we aim to crossbreed an Alzheimer mouse model (overexpressing APP with the Swedish-Dutch-Iowa mutations; APP_SweDI^13^) with GFP_FLT1 mice displaying green fluorescent protein (GFP^+^) in the endothelium of vessels^14^. This model may allow us to follow up Aβ plaques in close association with vessels. Our data will provide evidence that plaques develop with age. Importantly, already at early pre-plaque stage, Thiazin Red^+^ inclusions are found in brain GFP^+^ vessels. These inclusions represent aggregated platelets which may provide a first sign in the pathological cascade in AD.

## Results

### GFP^+^ vessels in APP_SweDIxGFP_FLT1 mice

**Figure 1:**
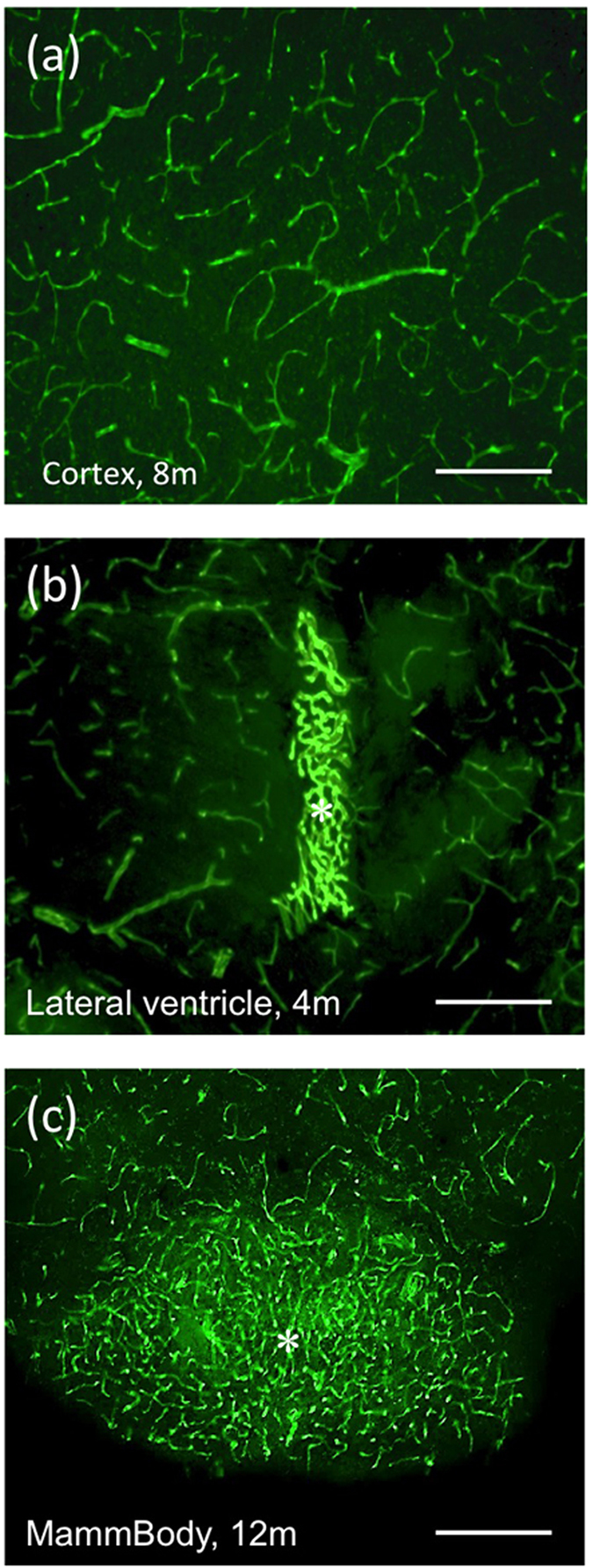
GFP^+^ vessels. Characterization of green fluorescent protein (eGFP^+^) vessels in the cortex of 8-month-old (**a**) crossbred APP_SweDIxGFP_FLT1 mice and in the lateral ventricle of 4-month-old mice (**b**, *) and in the mammillary body of 12-month-old mice (**c**, *). Brains were cryosectioned and directly visualized under the fluorescence microscope for GFP in the green channel (EX 480/40 nm, EM 527/30 nm). Scale bar: 115 μm (**a**–**c**).

A high number of GFP^+^ vessels was visible in all brain areas (cortex, hippocampus, thalamus, striatum, amygdala) of 4-months old mice (Fig. 1a; Table 1; see also Suppl. Fig. 1). The number of GFP^+^ vessels significantly decreased in 8 and 12-months old mice (Table 1). A very dense GFP^+^ vessel network was seen in the lateral ventricle (Fig. 1b) of 4-months (82 ± 6 optical density (OD), n = 5), 8-months (119 ± 4 OD, n = 6) and 12-months (124 ± 10 OD, n = 6) old mice. A higher density of vessels was also found in the mammillary body of 12-months old mice (Fig. 1c). The size of the vessels did not differ between 4 (13 ± 0.4 μm, n = 3), 8 (14 ± 0.6 μm, n = 3) or 12-months (12 ± 0.4 μm, n = 3) old crossbred mice.

### Thiazine Red^+^ staining in APP_SweDIxGFP_FLT1 mice

**Figure 2:**
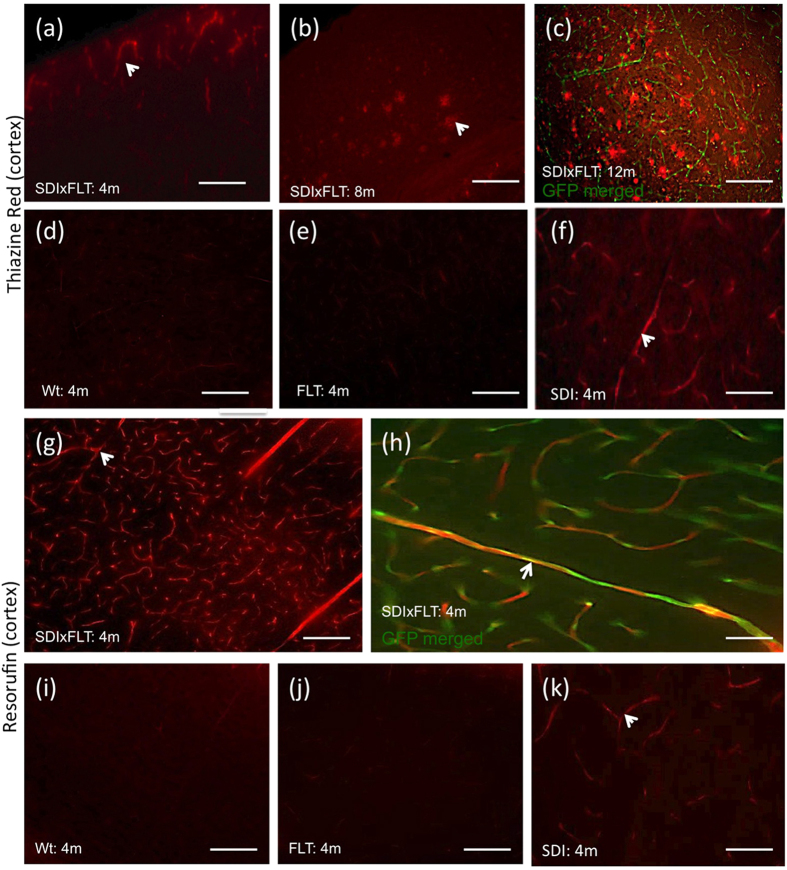
Thiazine Red^+^ and Resorufin stainings. Characterization of Thiazine Red (**a**–**f**) and Resorufin (**g**–**k**) stainings (arrows) in the cortex of 4 (**a**,**d**–**k**), 8 (**b**) and 12 (**c**) months old APP_SweDIxGFP_FLT1 (SDIxFLT) (**a**–**c**,**g**,**h**), wildtype (Wt) (**d**,**i**), GFP_FLT1 mice (FLT) (**e**,**j**) or APP_SweDI (**f**,**k**) mice. Brains were croysectioned and stained for Thiazine Red (**a**–**f**) or Resorufin (**g**–**k**) and visualized under the red channel (EX 535/50, EM 610/75). Note that in 4-months old crossbred mice no plaques but strong red vessel inclusions were found, while the number of plaques markedly increased in 8 and 12-months old mice. Note that the vessel inclusions were not seen in Wt and GFP_FLT1 mice, but in 4-months old APP_SweDI mice. Scale bar: 115 μm (**a**–**g**,**i**–**k**) and 70 μm (**h**). Abbreviation: Wt, wildtype; SDI, APP_SweDI; FLT, GFP_FLT1; SDIxFLT, APP_SweDIxGFP_FLT1.

In 4-months old mice very few Thiazine Red^+^ plaques were seen in hippocampus and lateral septum but more pronounced in the mammillary body (Table 2; Fig. 2a; see also Suppl. Fig. 1). A higher number of plaques was seen in 8 and 12-months old mice in all investigated brain areas (Fig. 2b,c; Table 2; see also Suppl. Fig. 3). However, in 4-months old non-perfused brains strong Thiazine Red^+^ inclusions were found in vessels (Fig. 2a). No Thiazine Red^+^ deposits were found in the lateral ventricle in 4–8 and 12-months old mice. No Thiazine Red^+^ stainings were seen in 4-months old wildtype (Wt) or GFP_FLT1 mice (Fig. 2d,e). However, in non-crossbred 4-months old APP_SweDI mice the same Thiazine Red^+^ inclusions were visible indicating that the inclusions are specific for the disease model (Fig. 2f).

### Resorufin staining in APP_SweDIxGFP_FLT1 mice

The vessel inclusions in 4-months old mice were verified by a Resorufin staining, which is another well-established marker for CAA. Strong red inclusions were found in GFP^+^ vessels (Fig. 2g,h). Again no stainings were seen in Wt and GFP_FLT1 mice (Fig. 2i,j). However again, in non-crossbred 4-months old APP_SweDI mice the same Thiazine Red^+^ inclusions were visible (Fig. 2k).

### Beta-amyloid stainings in APP_SweDIxGFP_FLT1 mice

**Figure 3:**
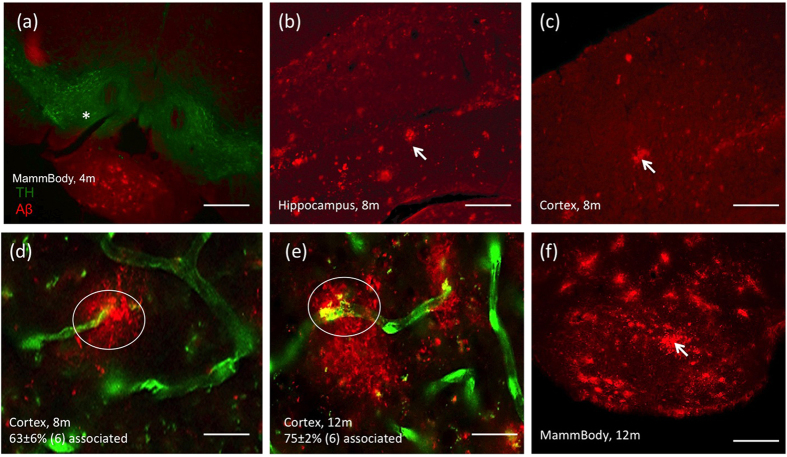
Beta-amyloid stainings. Characterization of beta-amyloid (Aβ) stainings in 4 (**a**), 8 (**b**–**d**) and 12 (**e**,**f**) months old APP_SweDIxGFP_FLT mice. Brains were transcardially perfused and croysectioned and stained for Aβ plaques (arrows), which was visualized under the red channel (EX 535/50, EM 610/75). The section containing the mammillary body (**a**, *) was counterstained with tyrosine hydroxylase (Alexa 488), which was visualized under the green channel (EX 480/40 nm, EM 527/30 nm). Green fluorescent protein (GFP, green) positive vessels were counterstained with Aβ (Alexa 546, red) and the number of associated structures (circles) was counted in 8 (**d**) and 12 (**e**) months old mice in the cortex. Note a high number of plaques in the 12-months old mammillary body (**f**). Scale bar: 300 μm (**a**), 115 μm (**b**,**c**,**f**) and 29 μm (**d**,**e**).

In accordance with Thiazine Red^+^ staining only few developing plaques were seen in the hippocampus, lateral septum and mammillary body (Fig. 3a; Table 2). The number of Aβ plaques markedly increased in 8-months old mice in the hippocampus (Fig. 3B) and cortex (Fig. 3c,d) and more pronounced in 12-months old mice (Fig. 3e; Table 2). Prominent Aβ plaques were found in the mammillary body of 12-months old mice (Fig. 3f). A high number of vessels was directly associated with Aβ plaques in 8 (63 ± 6%, n = 6; Fig. 3d) and 12 (75 ± 2%, n = 6, Fig. 3e) months old mice. No Aβ like immunoreactivity was found in the lateral ventricle in 4–8 and 12-months old mice.

### Thiazine Red^+^ inclusions in the confocal microscopy

**Figure 4:**
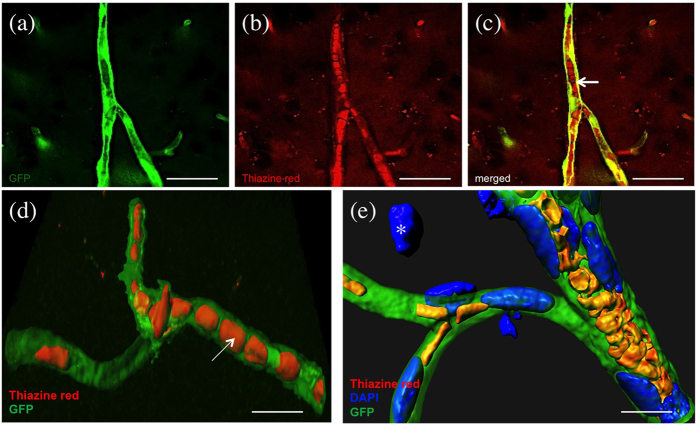
Thiazine Red inclusions in vessels. Thiazine Red^+^ inclusions in vessels in 4-months old non-perfused APP_SweDIxGFP_FLT mice. Brains were croysectioned and directly visualized under the fluorescence microscope for green fluorescent protein (GFP) (**a**,**c**–**e**) or stained for Thiazine Red (**b**–**e**). GFP was visualized under the green channel (EX 480/40 nm, EM 527/30 nm) and Thiazine Red in the red channel (EX 535/50, EM 610/75). (**c**) shows a merged picture with a red platelet in the vessel (arrow). High power microscopy was performed with confocal microscopy (**d**,**e**). Nuclei were counterstained with blue DAPI (**e**). Note several “red” platelets located within the “green” vessels (**d**, arrow). In **e** the contrast was increased in order to distinguish the different cellular structures. Note a nucleus located outside a vessel (**e**, *). Scale bar: 33 μm (**a**–**c**) and 5 μm (**d**,**e**).

Several pronounced Thiazine Red^+^ inclusions were found in GFP^+^ vessels in 4-months old non-perfused crossbred mice in the cortex and thalamus (Fig. 4a–c; Table 1). No such inclusions were seen in the hippocampus, striatum and amygdala (Table 1). Also no inclusions were seen in 8 and 12-months old mice (Table 1). These inclusions were also not found in perfused mice. Using confocal microscopy these inclusions were visualized as strong intracellular deposits in GFP^+^ vessels possibly representing blood cells (Fig. 4d,e).

### FACS analysis and aggregation

**Figure 5:**
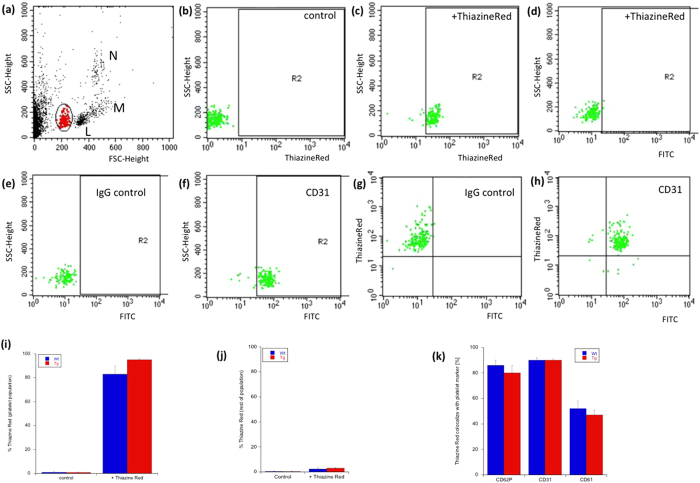
FACS analysis of Thiazine Red stained platelets. FACS analysis of whole EDTA mouse blood (**a**) shows several cell populations in the forward versus sideward scatter plot: neutrophils (N), monocytes (M) and leukocytes (L) as well as possibly platelets (red in circle). The platelet cell population was selectively stained with Thiazine Red (**c**), while controls were negative (**b**) and no signal was seen in the FITC channel (**d**). This cell population (n = 5) selectively stained for CD31 (**f**,**h**), CD62P and CD61 (**k**) compared to an IgG control (**e**,**h**). The platelet markers co-localized nearly to 100% with the Thiazine Red staining (**k**). No Thiazine Red was found in the other cell populations (**j**). Interestingly the Thiazine Red staining was not selective for the platelets of crossbred mice, but also seen in wildtype control mice (**i**,**j**).

Whole blood analysis defined several cell populations (Fig. 5a), including neutrophils, monocytes, leukocytes and possibly platelets. The platelet population was selectively stained with Thiazine Red (Fig. 5c), while controls were negative (Fig. 5b) and also no signal was seen in the FITC channel (Fig. 5d). This cell population was indeed identified as platelets, as they expressed CD31, CD62P and CD61 (Fig. 5e–k). The platelet markers co-localized to nearly 100% with the Thiazine Red staining (Fig. 5k). No Thiazine Red^+^ staining was visible in all other cell populations (Fig. 5j). However, the Thiazine Red labeling was not specific for the crossbred mice, because wildtype control mice (Fig. 5i) and GFP_FLT1 mice (data not shown) as well as 4-months old APP_SweDI mice (data not shown) also showed Thiazine Red stainings in platelets.

**Figure 6:**
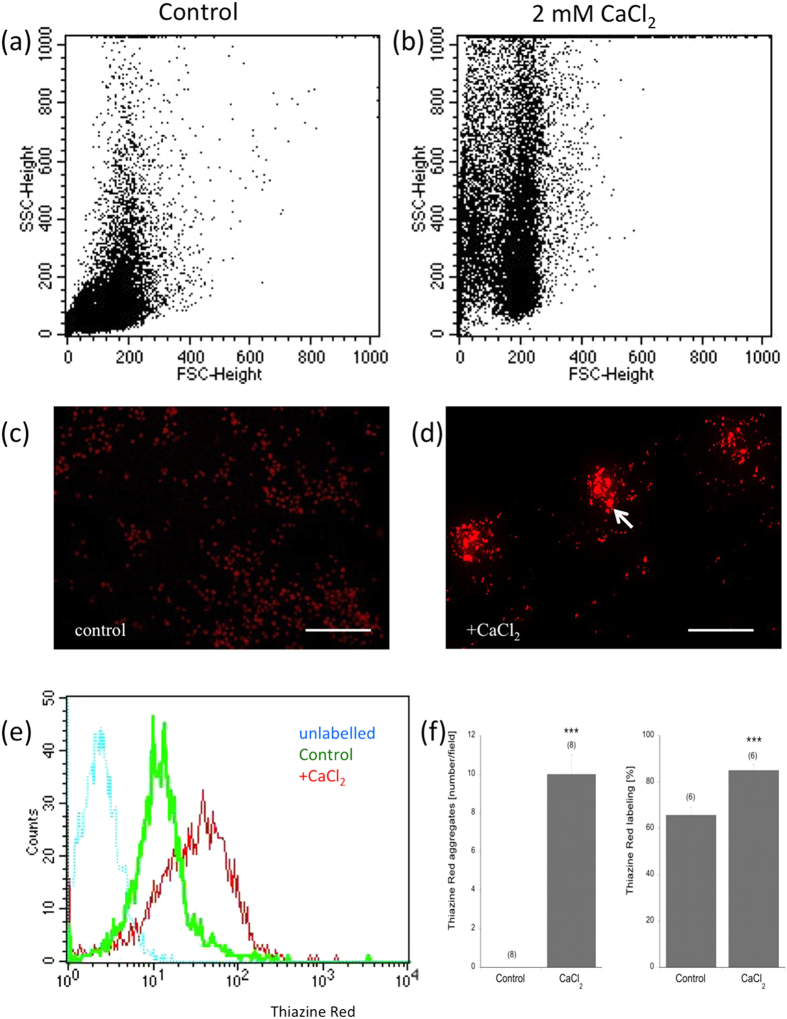
Aggregation of Thiazine Red^+^ stained platelets. Aggregation assay of isolated platelets in wildtype control mice. Platelets were isolated from EDTA blood, and incubated without (**a**,**c**) or with (**b**,**d**) 2 mM CaCl_2_ for 20 min at 37 °C, then fixed and stained with Thiazine Red (16 μg/ml, 2 hr, 4 °C). Note increased aggregation with 2 mM CaCl_2_ (**d** arrow, **f**) and enhanced Thiazine Red^+^ stainings (red) compared to controls (green) and unlabelled platelets (blue) (**e**). Values are given as mean ± SEM aggregates per field (10× magnification/13 × 18 cm) or % of labeling in the FACS. Statistical analysis was performed by Students T-test (***p < 0.001). Scale bar: 48 μm (**c**) and 96 μm (**d**).

A strong aggregation (8 ± 1 aggregates/field, n = 8) was seen in isolated platelets as visualized in the FACS after incubation with 2 mM CaCl_2_ for 20 min (Fig. 6a–d) compared to no aggregates in controls. The intensity of Thiazine Red^+^ staining was significantly enhanced (+19.3%, n = 6, compared to untreated platelets; Fig. 6f) in CaCl_2_-aggregated platelets as shown in the FACS further confirming Thiazine Red as a reliable marker for platelets (Fig. 6e).

## Discussion

In the present study we crossbred an Alzheimer mouse model (overexpressing amyloid precusor protein with the Swedish-Dutch-Iowa mutations, APP_SweDI) with mice showing green fluorescent protein eGFP^+^ endothelium in vessels (under the FLT-1 promoter; GFP_FLT1). This model allowed us to follow up plaques in close association with GFP^+^ vessels. Our data provide evidence that plaques develop with age, but at early pre-plaque stage/Thiazine Red^+^ inclusions are found in brain vessels. These inclusions represent aggregated platelets which may provide a first sign in the pathological cascade in AD.

### Beta-amyloid plaques in AD

The APP_SweDI (Swedish/Dutch/Iowa) transgenic mouse model was first described by Davis *et al*.^13^. These transgenic mice exhibit a strong Aβ-associated pathology and neurotoxic plaque formation in the wall of brain blood vessels representing CAA. However, this model does not show any Tau pathology. In the present study we confirm that no or few Aβ plaques are present in 4-months old mice but markedly progress with aging being most prominent at 12-months of age. The plaques were verified by highly specific immunostainings for Aβ using the 6E10 monoclonal antibody. These findings were also confirmed using Thiazine Red^+^ which is a marker to differentiate the fibrillar state from the nonfibrillar state of Aβ in AD^15^,^16^. The Aβ plaques were very prominent in all brain areas in 12-month-old mice. However, the mammillary bodies were among the first brain regions showing plaques. Indeed, recent findings suggest that the mammillary bodies may play a role in memory that is independent of their hippocampal formation afferents. It has been indicated that the medial mammillary nucleus is predominantly responsible for spatial memory deficits^17^. In addition, early Aβ plaques also appeared in the lateral septal nucleus, which is a brain region containing cholinergic neurons. In fact, a decline of cholinergic neurons and the neurotransmitter acetylcholine is directly linked to cognitive impairment. However, the mechanism whereby Aβ induces cholinergic cell loss and cognitive impairment remains obscure^18^.

### Vessel pathology in AD

The GFP_FLT1 mouse model directly allows to visualize vessels in the brain. In fact, a strong GFP^+^ network was seen throughout the brain in young mice and this vascular network significantly declined during progression of Aβ plaque development. Our data show, that Aβ plaques were clearly associated with GFP^+^ vessels. The central question arises: What are the reasons that the GFP^+^ vessel network is markedly reduced. Four causes may account for this observation: (1) The vascular decline is a direct cause of the inflammatory processes during progression of AD, (2) the Aβ plaque depositions directly induce vessel alterations, (3) the GFP^+^ fluorescence just simply decreases with age of the animals or (4) the GFP^+^ protein directly causes toxicity of the vessels during the AD progression. First, it is well established that microvascular degeneration, fragmented vessels, microvessel inflammation and microinfarcts are commonly observed in AD brains and are known to disrupt the integrity of cerebral vessels^6^,^19^,^20^. It is known, that changes in capillary diameter and density are related to aging and that capillary density decreases in AD^21^,^22^. Further the basement membrane of cerebral vessels are altered and thickened^21^. Inflammatory factors such as IL-1β, IL-6 or TNF-α are elevated in microvessels in an AD brain^23^ and may play a role in the progression of AD. Our data show, that the vascular network in our crossed mouse model significantly declined during progression of Aβ plaque development, however, we neither detected inflammatory processes nor other vessel specific markers (e.g. laminin) were used to verify the decline of vessels during AD progression. Second, it is known, that Aβ-associated CAA is the most common vascular lesion in AD and the majority of Aβ peptides are formed on the surrounding vasculature^24^. Moreover, the Aβ aggregates in vessels perturb BBB integrity^6^ and thus may cause degeneration of vessels and cerebral capillaries^24^,^25^. Furthermore, an autopsy study of AD patients showed that a majority of Aβ plaques are highly associated with blood vessels^26^. Our data are in line and show a high association of Aβ plaques with vessels. It seems reasonable that Aβ deposition in vessels may interact with GFP and may induce vessel degeneration. Third, in the GFP_FLT1 mouse model^8^, GFP is highly expressed in vessels driven by the vascular endothelial growth factor receptor VEGFR-1 (FLT-1) promotor and this allows to follow up the vessel pathology in our crossbred mice. It seems possible that during aging an altered FLT1 promotor may cause a downregulation of GFP^+^ protein expression. Furthermore, an interaction between Aβ deposition in plaques or vessels may further potentiate such an inactivation of this promoter. Fourth, there is clear evidence that GFP expression for extended periods may generate free radicals which are toxic to cells^27^,^28^ while Okabe *et al*.^29^ did not show any toxicity in a mouse model expressing GFP. Thus, in our study we cannot exclude that GFP is toxic to the vessels causing vessel decline. As this is very unlikely, it seems possible that the Aβ-induced vessel deposition may potentiate GFP^+^ toxicity in our crossbred mice. Further investigation of the causes of GFP^+^ vessel decline are necessary but not in the focus of this study.

### Thiazine Red^+^ inclusions in 4-months old non-perfused mice

Thiazine Red and Thioflavin S are well-established histochemical markers of dense core plaques. Thiazine Red is a marker to differentiate the fibrillar from the nonfibrillar state of Aβ in AD^15^. Thiazine Red shows analogy to naphtol-based azo structures whose functional characteristic is to bind β-sheet structures, while providing a site to complex ferrous ion^30^. Moreover, Thiazine Red binds to several beta-sheet aggregates including Aβ, Tau or alpha-synuclein. Our data show that indeed Thiazine Red was useful to stain plaques and their number highly correlated to the number of Aβ plaques. However, in addition we observed several Thiazine Red^+^ inclusions in brain vessels. These inclusions were only found in non-perfused brains, pointing to blood-derived cells. Further these inclusions were only seen in 4-months old crossbred mice but not in older animals and also not in wildtype or not-crossed mice. Additionally, we also observed the same Thiazine Red^+^ inclusions and Resorufin+ inclusions in 4-months old non-crossbred APP_SweDI mice. This gives clear evidence that these signs are a typical pathological signature for early AD and not only a consequence of the crossbreeding or of degenerating vessels. In addition, these inclusions also did not stain for DAPI, and thus did not contain a nucleus, further pointing to anuclear cells. Thus our first evidence suggests that the Thiazine Red^+^ inclusions represent platelets.

### Resorufin stained vessels and CAA

CAA is characterized by deposition of Aβ peptides in the walls of leptomeningeal and parenchymal vessels^31^ and therefore a feature of aging and AD. Furthermore, CAA is also associated with intracerebral hemorrhage^32^ caused by Aβ accumulation in the leptomeningeal and intracranial arteries with smooth muscle loss, leading to necrosis, rupture as well as aneurysm formation. However, several investigations established that both soluble and insoluble forms of Aβ in particular the CAA associated Aβ_40_ were strongly vasoactive^33^. Furthermore it is known, that CAA induces degeneration as well as destruction of the vessel wall and affects cerebral blood flow^9^ which is an early feature of AD^33^. Based on these findings, an open question related to CAA concerns the origin or source of Aβ peptides in cerebral vessels. Moderate to severe Aβ deposition in the walls of arterial blood vessels leads to thickening of the vessel wall that can be seen in haematoxylin and eosin stained sections. Hence, histochemical (Congo Red, Thioflavin S) or immunohistochemical Aβ staining is needed to detect CAA^11^. Further, in order to characterize CAA Resorufin staining is well known^8^ because the phenoxazine derivative Resorufin preferentially binds cerebrovascular amyloid deposits. This unique binding suggests that Resorufin has an excellent selective potential as a CAA specific dye that will permit a detection as well quantification of CAA^8^. We observed, that the phenoxazine derivative Resorufin binds peptides of CAA in a highly specific manner in 4-months old APP_SweDIxGFP_FLT1 mice. It seems likely, that microvasculature alterations occur at younger ages, when typically parenchymal amyloid plaques are not yet present^6^. In summary, our data may suggest vessel deformation and small amyloid deposits in the vessel walls being in line with others^6^,^7^. Therefore, the accumulation of Aβ-associations with blood vessels causing CAA may play a critical role in the development of AD.

### Thiazine Red^+^ inclusions and platelets

In order to characterize the Thiazine Red^+^ staining in platelets we performed FACS analysis. We show for the first time that platelets can indeed be stained with Thiazine Red compared to other blood cells. On the other hand we observed that also platelets from 4-months old wildtype mice, GFP_FLT1 mice as well as APP_SweDI mice were stained with Thiazine Red^+^. Therefore, the Thiazine Red^+^ staining is not specific for our mouse model, where we crossbred APP_SweDI with GFP_FLT1 mice. While Thiazine Red is an accurate marker to differentiate the fibrillar from the nonfibrillar state of Aβ in AD^16^ we cannot prove yet the exact binding site of Thiazine Red^+^ in platelets. However it is known, that platelets contain high amounts of APP and produce small amounts of Aβ, predominantly Aβ_40_ over Aβ_42_ (ref 10) Thus it seems likely that Thiazine Red binds Aβ or APP in platelets.

Interestingly, increasing evidence suggests that platelet activation can also mediate the onset and development of CAA, because activated platelets express and release more than 90% of the circulating peripheral Aβ (mainly Aβ_40_) which in turn activates platelets and results in the vicious cycle of Aβ overproduction in damaged vessel^10^,^33^,^34^,^35^. Interestingly Roher *et al*.^36^ reported that platelets release Aβ when they come in contact with soluble exogenous Aβ that is also present in blood under physiological and pathophysiological conditions. Furthermore, platelets are able to transfer soluble Aβ into fibrillar structures which might contribute to CAA. As platelets are crucial for hemostasis at sites of vascular injury, uncontrolled platelet activation may cause acute vessel occlusion which may lead to alterations in blood flow^9^ and such decreased cerebral blood flow negatively affects brain metabolism and synthesis of proteins required for memory and learning^33^. As we observed that activated and consequently aggregated platelets display increased Thiazine Red^+^ staining, it is hypothesized that platelets may aggregate early in AD and form the Thiazine Red^+^ inclusions.

In conclusion, our data show for the first time that Thiazine Red^+^ inclusions representing aggregated platelets are a first pathological sign in AD before plaque development. Therefore, we hypothesize, that dysregulated platelets have an important influence on the progression of AD and may become important therapeutic targets in early AD.

## Methods

### Mouse models

The Alzheimer mouse model (C57B1/6-Tg (Thy1-APPSweDuIowa)Bwevn/Mmjax, APP_SweDI) was obtained from MMRRC (USA). These transgenic mice express neuronally derived human amyloid beta-precursor protein (APP 770 isoform) with the Swedish K670N/M671L, Dutch E693Q and Iowa D694N mutations (APP_SweDI). This model has been fully characterized and exhibits marked Aβ plaques in brain and vessels after 6-months of age^13^. The green fluorescent protein (GFP) vessel mice have been fully characterized by Herz *et al*.^14^. The GFP is under control of the FLT1 (FMS-like tyrosine kinase 1) promotor and these mice express GFP in nearly all vessels (GFP_FLT1 mice). APP_SweDI and GFP_FLT1 mice were crossbred under standard conditions. All animal experiments were approved by the Austrian Ministry of Science and Research (BMWF-66.011/0044-II/3b/2011 and BMWF-66.011/0059-II/3b/2011) and conformed to the Austrian guidelines on animal welfare and experimentation. All possible steps were taken to reduce suffering and the number of animals used during the experiment.

### Processing of brains

Four, 8 and 12-months old APP_SweDI x GFP_FLT1 mice were used. (1) Non-perfused brains: Mice were anesthetized by Ketamine 100 mg/kg and Xylazine 10 mg/kg (AniMedica) and the brains removed. One hemisphere was frozen on a cork in a CO_2_ stream. The other hemisphere was fixed overnight in 4% paraformaldehyde (PAF) in 10 mM phosphate-buffered saline (PBS), and then cryoprotected overnight in 20% sucrose and then frozen in a CO_2_ stream. The brains were stored at −80 °C until use. (2) Perfused brains: Anesthetized mice were transcardially perfused with 50 ml PBS containing heparin and Ethylenediaminetetraacetic acid (EDTA) and then with 50 ml of 4% paraformaldehyde (PAF) in PBS. Brains were removed, postfixed for 3 hours in 4% PAF, then cryoprotected in 20% sucrose overnight and stored in PBS/NaAcide until use. Brains were sectioned into 40 μm sections with a cryostat (Leica CM 1950).

### Immunohistochemistry

The brain sections were washed with PBS and incubated in PBS/0.1% Triton (T-PBS) for 30 min at 20 °C while shaking. After incubation, the sections were blocked in T-PBS, 20% horse serum (Gibco Invitrogen) for 30 min at 20 °C while shaking. Following blocking, brain sections were incubated with primary Aβ antibody (Aβ, 1–16 (6E10), Covance, 1:1000) in T-PBS and 0.2% bovine serum albumin (BSA) for 2–3 days at 4 °C. The sections where then washed and incubated with anti-mouse fluorescent Alexa 546 antibody (Invitrogen-Life tech, Vienna, Austria) in T-PBS and 0.2% BSA for 1 hour at 20 °C while shaking. Finally the sections were washed and then mounted onto glass slides and coverslipped with Mowiol^®^ 4–88 (Roth, Austria). Some sections were stained with Thiazine Red or Resorufin (1.6 μg/ml, Sigma, overnight) to label plaques or CAA respectively. Some sections were counterstained with tyrosine hydroxylase antibodies (Novus, NB300-109; 1:1000, secondary anti-rabbit Alexa 488). Some sections were counterstained with blue DAPI (1:10 000, 1 hour) to visualize nuclei.

### Quantification of Fluorescence Staining

The number of Aβ plaques and GFP^+^ vascular network was counted under the fluorescence microscope (2D images) (Olympus, Bx61) at a 10x magnification. Alexa 546 and Thiazine Red/Resorufin were visualized under the red filter Y3 (EX 535/50 nm, EM 610/75 nm), eGFP under the green filter L5 (EX 480/40 nm, EM 527/30 nm). Confocal microscopy was performed using SP5 confocal microscope (3D images) (Leica Microsystems, Wetzler, Germany) with an HCX PL APO x63 and/or 1.3 NA glycerol objective. Images were aquired using the LAS AF aquisition sofware, version 2.1, and further processed with Huygens Deconvolution and Imaris 8.1 Image management software. Confocal imaging was performed with an argon laser line (set power to 20%) for AlexaFluor 488, a DPSS561 nm laser for AlexaFluor 546 or Thiazine Red and a 405 diode laser for DAPI. Emission of each fluorophore was detected from 493 to 556 nm (AlexaFluor 488, eGFP), 566 to 628 nm (AlexaFluor 546, Thiazine Red) and 418 to 483 nm (DAPI). For the control panel the smart gain was set to 250 Volt (V) per turn, smart offset to 0.1 or 1%, zoom to medium, X position to fine, Y position to fine and the resolution was set to 12 bit, pixels size between 40 and 60 pixels, speed to approximately 1000 Hertz (Hz), frame resolution to 1024 × 1024 and the line average between 1–3. General parameters for the sampling intervals was set to X (nm) 60.125, Y (nm) 60.125, Z (nm) 125.885. For the objective correction the Photomultiplier (PMT) was activated and set to a gain of 500–600 V and the Scan Mode from XYZ to XZY. Afterwards AOBS was clicked and the setting changed to Reflection. The PMT detector range was set to min 487 nm and max 556 nm and “between lines” in the scanning mode. For the Deconvolution with the Huygens software the following parameters were set: numerical aperture (1.3), objective quality (good), coverslip position (μm), imaging directions (upward), lens immersion (glycerine, 1.474), embedding (Glyc. 90%, Mowiol, 1.458), backprojecting pinhole (307.09 nm), excitation fill factor (2.00), signal/noise per channel (15,15,15), max iterations (100), the search for background (auto), the background per channel (0.0, 0.0., 0.0), bleaching correction (if possible), brick mode (auto), quality change threshold (0.1%), iteration mode (optimized) and padding mode (automatic). After the Deconvolution the images were processed with the Imaris 8.1 sofware for 3D imaging.

### FACS analysis and aggregation assay

Mouse platelets were isolated as reported previously^12^. Briefly, mice were anaesthetized by an intraperitoneal injection of Ketamine 100 mg/kg and Xylazine 10 mg/kg (AniMedica). Blood drawn directly from the heart was immediately collected in ethylenediaminetetraacetate (EDTA) tubes (S-monovettes, Sarstedt, Germany). Immediately after isolation, 5 μl EDTA blood was mixed with 95 μl FACS buffer (2 mM EDTA, 0.5% FCS ad 100 ml PBS, pH 7.1) and 16 μg/ml Thiazine Red and incubated for 3 hours at 4 °C. Then the cells were lysed using Miltenyi red blood cell lysis buffer, centrifuged and the pellet dissolved in 100 μl FACS flow. Some samples were incubated just prior the lysis with 5 μl of IgG1-FITC control (Miltenyi 130-089-867), CD62P-FITC (BD Biosciences, Heidelberg, Germany, Cat: 561923), CD31-FITC (Miltenyi 130-097-424) or CD61-FITC (Miltenyi 130-098-722) for 15 min at room temperature. FACs analysis was instantly performed with a BD FACScan. For the aggregation assays, isolated platelets were incubated with or without 2 mM CaCl_2_ for 20 min at 37 °C, then fixed with 4% PAF, and incubated with 16 μg/ml Thiazine Red for 2 hours at 4 °C, cells were centrifuged and visualized under the microscope or in the FACS.

### Data analysis and statistics

Plaques were identified either by Thiazine Red^+^ staining or using immunohistochemistry for Aβ. GFP^+^ vessels were visualized using the green channel (EX 480/40 nm, EM 527/30 nm) and Thiazine Red^+^ inclusion, Resorufin staining or Alexa 546^+^ plaques in the red filter Y3 (EX 535/50 nm, EM 610/75 nm). The number of plaques was counted under the microscope at a 10× magnification by a blinded observer (10 plaques per cortex 5 sections per brain). The number of vessel crossings was counted in a 6 × 6 grid. The number of vessels associated with plaques was counted when the vessels were directly connected to a plaque. The optical density of the lateral ventricle was measured by computer-assisted imaging using the histogram function of ImageJ. Statistical analysis was performed by One way analysis of variance (ANOVA) and subsequent Fisher least significant difference post hoc test (*p < 0.05; **p < 0.01; ***p < 0.001; ns not significant).

## Additional Information

**How to cite this article**: Kniewallner, K. M. *et al*. Thiazine Red^+^ platelet inclusions in Cerebral Blood Vessels are first signs in an Alzheimer’s Disease mouse model. *Sci. Rep.* **6**, 28447; doi: 10.1038/srep28447 (2016).

## Supplementary Material

Supplementary Information

## Figures and Tables

**Table 1:** Quantification of GFP^+^ vessels in 4, 8 and 12-month-old mice APP_SweDIxGFP_FLT mice.

	GFP^+^ Vessel crossings			% Thiazine Red inclusions in GFP^+^ vessel		
	4 mo	8 mo	12 mo	4 mo	8 mo	12 mo
Cortex	127 ± 2 (10)	63 ± 2 (6)***	44 ± 2 (6)***	19 ± 2 (10)	0 (6)***	0 (6) ns
Hippocampus	83 ± 3 (10)	37 ± 1 (6)***	32 ± 1 (6)***	0 (10)	0 (6) ns	0 (6) ns
Thalamus	141 ± 2 (10)	72 ± 1 (6)***	57 ± 1 (6)***	14 ± 6 (10)	0 (6)***	0 (6) ns
Striatum	90 ± 2 (10)	56 ± 1 (6)***	41 ± 1 (6)***	0 (10)	0 (6) ns	0 (6) ns
Amygdala	128 ± 3 (10)	67 ± 3 (6)***	30 ± 0.5 (6)***	0 (10)	0 (6) ns	0 (6) ns

Brains of 4–8–12-months (mo) old APP_SweDIxGFP_FLT1 mice were fresh frozen under a CO_2_ stream, cryosectioned (40 μm), counterstained with Thiazine Red and the GFP^+^ vessels were quantified in 5 brain areas at a 10× magnification (4 mo 5 sections/brain, 8 mo 5 sections/brain, 12 mo 5 sections/brain; Suppl. Fig. 2B). The number of vessel crossings was counted in a 6 × 6 grid (Suppl. Fig. 2A). The % Thiazine Red^+^ vessels were measured by fluorescence microscopy in the red filter (EX 535/50, EM 610/75). The field was photographed in the green channel (EX 480/40, EM 527/30) and then the Thiazine Red^+^ vessels photographed in the red filter. Pictures were merged and the % of Thiazine Red^+^ vessels were counted. Values are given as mean ± SEM, the values in parenthesis give the number of analyzed animals. Statistical analysis was performed by comparing 4 versus 8 and 8 versus 12-month-old mice using one Way ANOVA with a subsequent Fisher LSD posthoc test (***p < 0.001; ns not significant).

**Table 2:** Quantification of plaques in 4,8 and 12-month-old mice APP_SweDIxGFP_FLT1 mice.

	Thiazine Red+plaques			Aβ+ plaques		
	4 mo	8 mo	12 mo	4 mo	8 mo	12 mo
Cortex	0 (15)	26 ± 2 (6)***	36 ± 1 (6)**	0 (10)	20 ± 2 (6)***	39 ± 0.6 (6)***
Hippocampus	6 ± 1 (15)	20 ± 2 (6)***	26 ± 1 (6)**	4 ± 1 (10)	21 ± 1 (6)***	43 ± 0.4 (6)***
Thalamus	0 (15)	8 ± 1 (6)***	19 ± 1 (6)***	0 (10)	13 ± 1 (6)***	25 ± 0.5 (6)***
Striatum	0 (15)	2 ± 0 (6)***	13 ± 1 (6)***	0 (10)	4 ± 0 (6)***	13 ± 0.7 (6)***
Amygdala	0 (15)	23 ± 1 (6)***	34 ± 0.8 (6)***	0 (10)	24 ± 0 (6)***	46 ± 1 (6)***
Mammillary body	12 ± 2 (15)	21 ± 1 (6)***	32 ± 1 (6)***	8 ± 4 (10)	17 ± 2 (6)ns	31 ± 1 (6)***
Lateral septal nucleus	3 ± 4 (15)	15 ± 2 (6)**	28 ± 0.5 (6)***	4 ± 4 (10)	13 ± 1 (6)*	29 ± 1 (6)***

Animals (4–8–12-months, mo) were transcardially perfused with paraformaldehyde, the brains immersed in sucrose, and then frozen and cryosectioned (40 μm). Brain sections were either stained by Thiazine Red or using immunohistochemistry for beta-amyloid (Aβ). The number of plaques was counted under the microscope at a 10× magnification (Thiazine Red^+^ plaques 5 sections/brain, Aβ Immunohistochemistry 5 sections/brain in 4-month and 8-month-old mice and 12-months old mice). Values are given as mean ± SEM, the values in parenthesis give the number of analyzed animals. Statistical analysis was performed by comparing 4 versus 8 and 8 versus 12-months old mice using one Way ANOVA with a subsequent Fisher LSD posthoc test (*p < 0.05; **p < 0.01; ***p < 0.001; ns not significant).
