# Supplementary material for: Thiazine Red+ platelet inclusions in Cerebral Blood Vessels are first signs in an Alzheimer’s Disease mouse model
Source: Sci Rep. 2016 Jun 27;6:28447. doi: 10.1038/srep28447 (PMC4921929; doi:10.1038/srep28447)
Supplement: Supplementary Information [file srep28447-s1.pdf]

# Thiazine Red$^+$ platelet inclusions in Cerebral Blood Vessels are first signs in an Alzheimer's Disease mouse model

Kathrin M. Kniewallner, Daniela Wenzel and Christian Humpel

## SUPPLEMENTARY FIGURE LEGENDS

Supplementary Figure 1: Overview of the 4 month old APP_SweDIxGFP_FLT1 mice. Perfused and non-perfused brains were cryosectioned and directly visualized under the fluorescence microscope (2D images). Brain sections were immunohistochemically stained with Thiazine Red. Green fluorescent protein (GFP) was visualized under the green channel (EX 480/40 nm, EM 527/30 nm) and Thiazine Red under the red channel (EX 535/50, EM 610/75). Fig. **a** shows the brain areas we analyzed (filled red box). Note, several GFP$^+$ brain vessels in all analyzed brain areas (**b**). Fig. **c** shows Thiazine Red APP_SweDIxGFP_FLT1 perfused brain sections (4 month). Note, Thiazine Red plaques were only visible in the hippocampus, mammillary body and the lateral septum (**c** perfused, arrows). Fig. d shows Thiazine Red inclusions in vessels in 4 month old APP_SweDIxGFP_FLT1 non-perfused brains. Note, several Thiazine Red inclusions in the cortex and thalamus (**d** non-perfused, arrows).

Supplementary Figure 2: Evaluation of vascular green fluorescent protein (GFP$^+$) vessels and beta-amyloid (Aβ) plaques in cortex brain sections. GFP was visualized under the green channel (EX 480/40 nm, EM 527/30 nm) (**a**) and Thiazine Red/Aβ-Alexa546 was visualized under the red channel (EX 535/50, EM 610/75). Fluorescence images were aquired using the Openlab software (4.0.4) (**a&b**).The vascular network of GFP$^+$ vessel was quantified by counting the number of vessel crossings in a 6x6 grid (**a**, red dots, 121 counts, 10x magnification). The number of Aβ plaques was counted under the fluorescence microscope (**b**, white circles, 21 counts, 10x magnification).

Supplementary Figure 3: Analysis of 4, 8 and 12 month old non perfused APP_SweDIxGFP_FLT1 mice in the cortex. Brains were cryosectioned and directly

visualized under the fluorescense microscope (2D images). Brain sections were immunohistochemically stained either with beta-amyloid Alexa546 or Thiazine Red and were visualized under the red channel (EX 535/50, EM 610/75). Fig. **a** shows pictures from 4, 8 and 12 month old brain sections in a 10x (**a**) or 60x (**b**) magnification immunohistochemically stained with beta-amyloid Alexa 546. Note, several plaques in 8 and 12 month old mice (**a**). Fig. **c** (10x mag) &**d** (60x mag) shows Thiazine Red plaques in the cortex of 4, 8 and 12 month old APP_SweDIx GFP_FLT1 mice. Note, several Thiazine Red plaques in 8 and 12 month old mice (**c**). Fig. **e** (10x mag) & **f** (60x mag) shows Thiazine Red inclusions in 4 month old non perfused APP_SweDIxGFP_FLT1 mice compared to older mice.

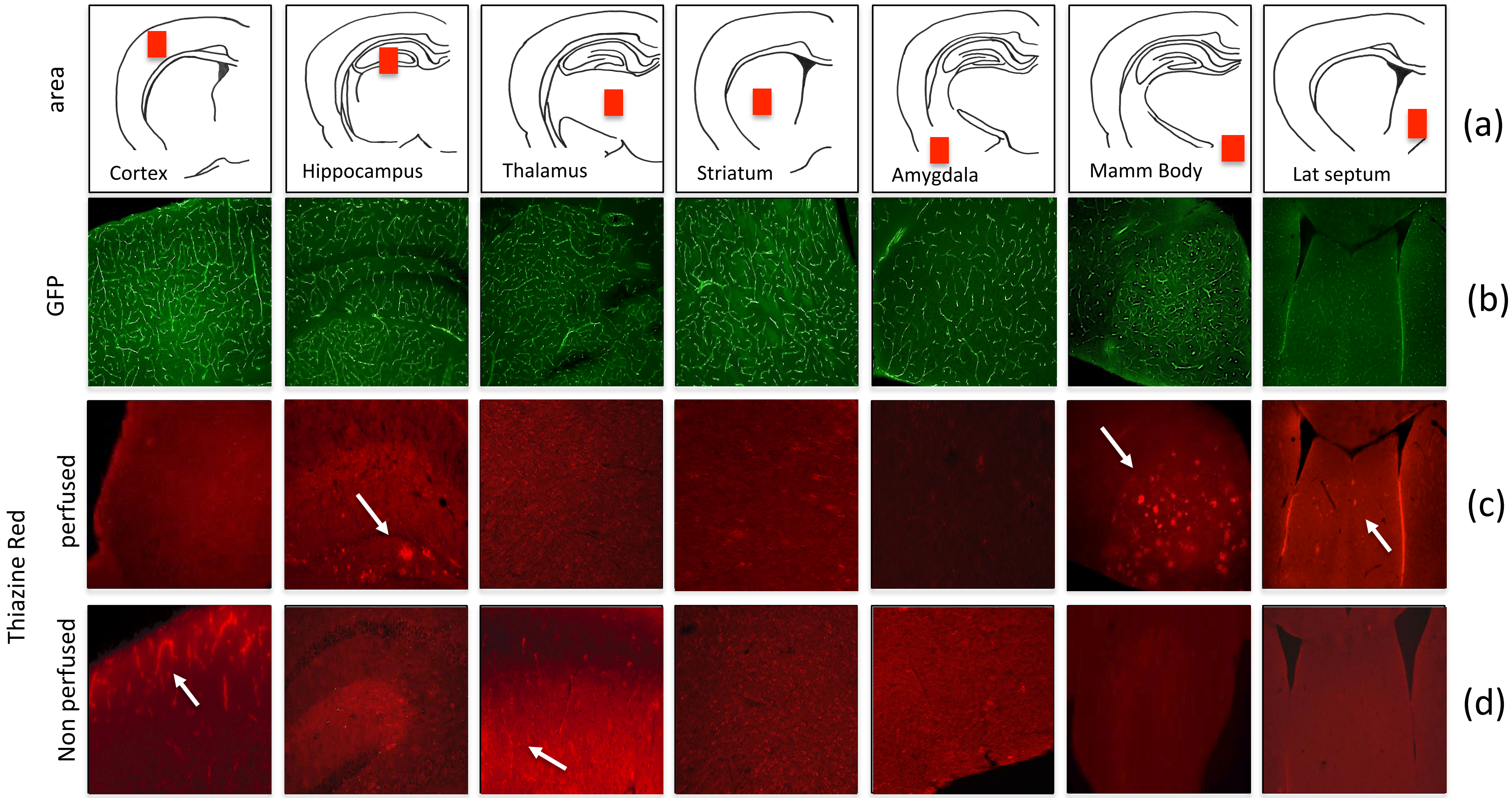

area
Cortex
Hippocampus
Thalamus
Striatum
Amygdala
Mamm Body
Lat septum
(a)
GFP
(b)
Thiazine Red
perfused
(c)
Non perfused
(d)

(a)

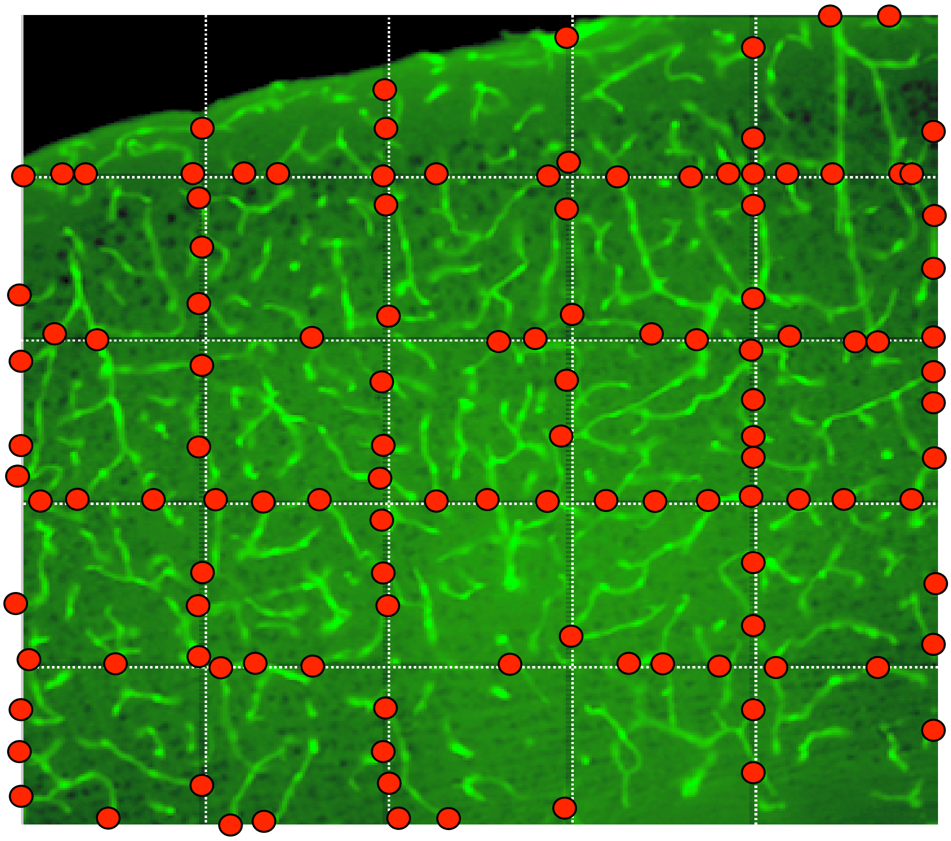

(b)

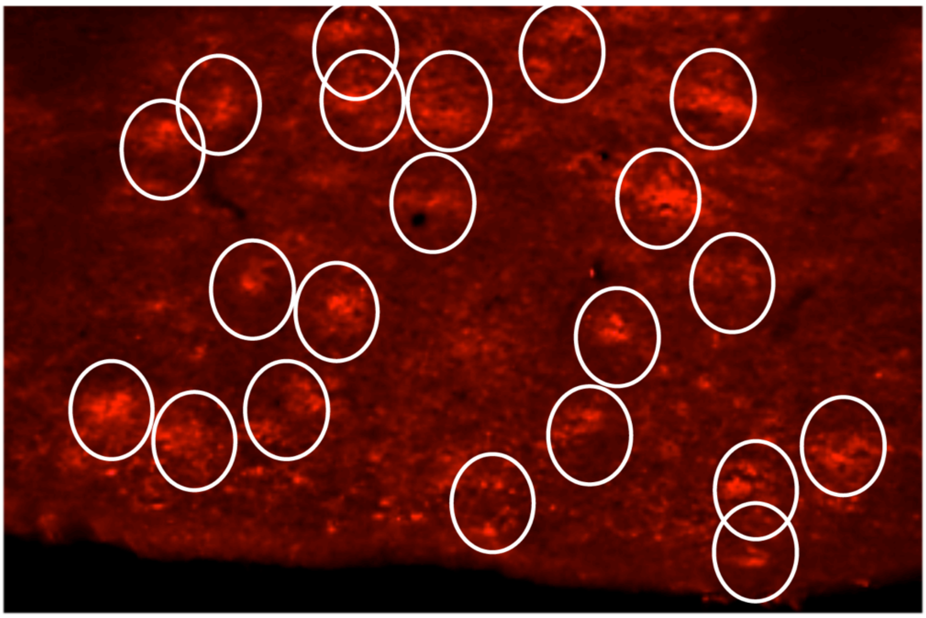

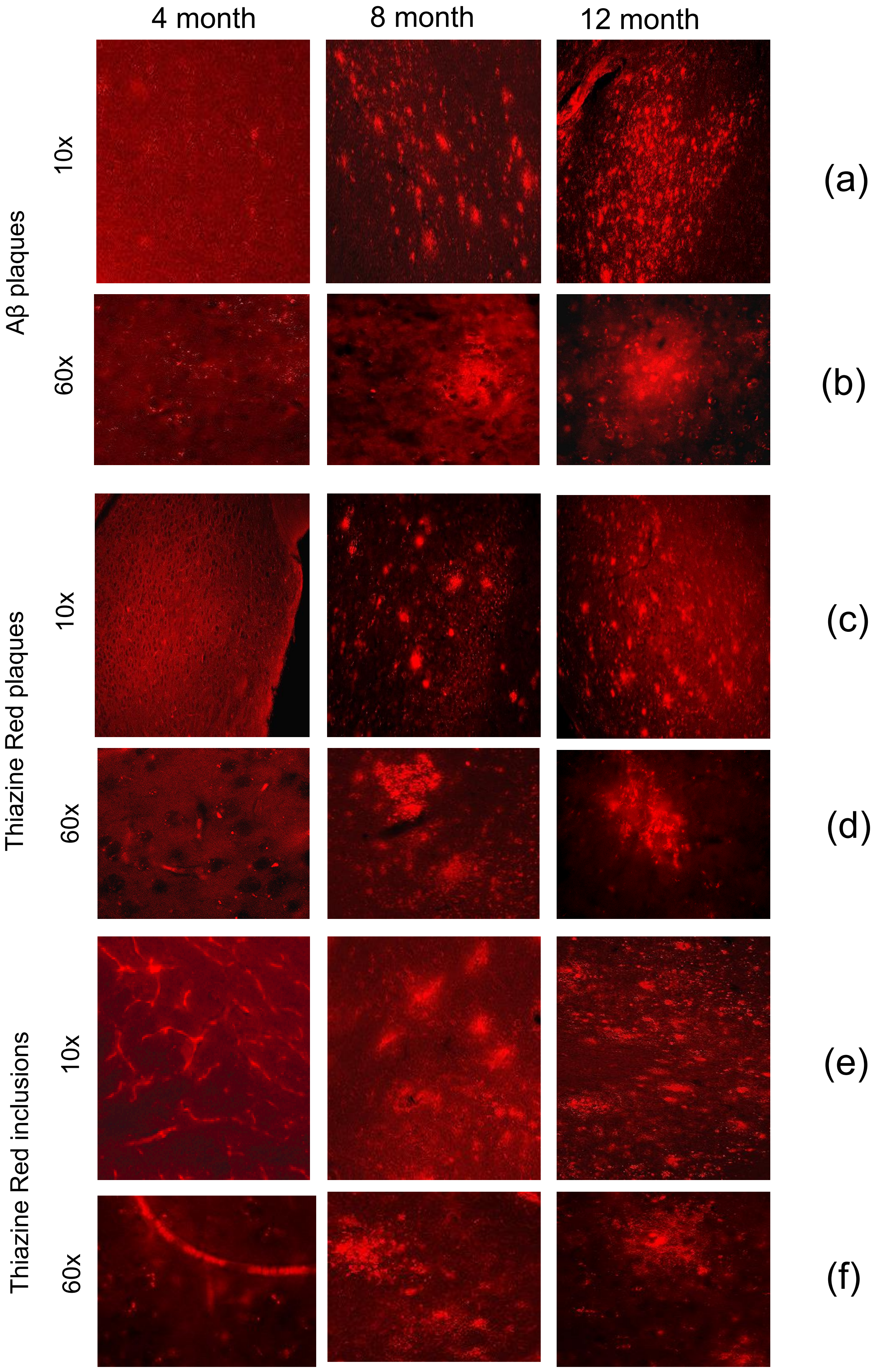

4 month
8 month
12 month
Aβ plaques
10x
60x
Thiazine Red plaques
10x
60x
Thiazine Red inclusions
10x
60x
(a)
(b)
(c)
(d)
(e)
(f)
